# Horseshoe Kidney Isthmus Infarction After Percutaneous Endovascular Aortic Aneurysm Repair

**DOI:** 10.7759/cureus.7279

**Published:** 2020-03-15

**Authors:** Patrick D Melmer, Akash Patel, Saptarshi Biswas, Mark R Borowicz

**Affiliations:** 1 Surgery, Grand Strand Medical Center, Myrtle Beach, USA; 2 Surgery, Grand Strand Medical Center/University of South Carolina, Myrtle Beach, USA; 3 Vascular Surgery, Grand Strand Medical Center/University of South Carolina, Myrtle Beach, USA

**Keywords:** horseshoe, kidney, evar, pevar

## Abstract

Horseshoe kidneys and their wide-ranging anatomy present a unique test to the open surgical approach for repair of abdominal aortic aneurysms. Endovascular options are currently the desired strategy for treatment of abdominal aortic aneurysms and offer multiple advantages when horseshoe kidneys are present. Even so, the challenging nature of these patients demands a high degree of caution and planning. This case details a patient with horseshoe kidney who underwent percutaneous endovascular aneurysm repair for an abdominal aortic aneurysm and developed isthmus infarction early in the postoperative period with an uneventful subsequent recovery. Here we report what we believe is the first successfully described percutaneous endovascular aneurysm repair to treat a patient with horseshoe kidney.

## Introduction

Abdominal aortic aneurysms (AAAs) in the setting of associated horseshoe kidneys (HSK) present a unique challenge to the vascular surgeon. Endovascular aneurysm repair (EVAR) has emerged as the desired approach for AAA treatment when technically feasible, and offers further advantages in the setting of HSK due to the ability to avoid isthmus resection, collecting system damage, or other disturbances to the renal system. Percutaneous endovascular aneurysm repair (PEVAR) adds to this with further minimally invasive advances. EVAR has been shown to be an effective strategy with good outcomes for patients with HSK [1,2]. Even so, extreme caution should be undertaken given the challenging nature of these patients. We present a case of a patient who underwent PEVAR for a AAA with associated HSK and developed isthmus infarction early in the postoperative period with an uneventful subsequent recovery.

## Case presentation

**Figure 1:**
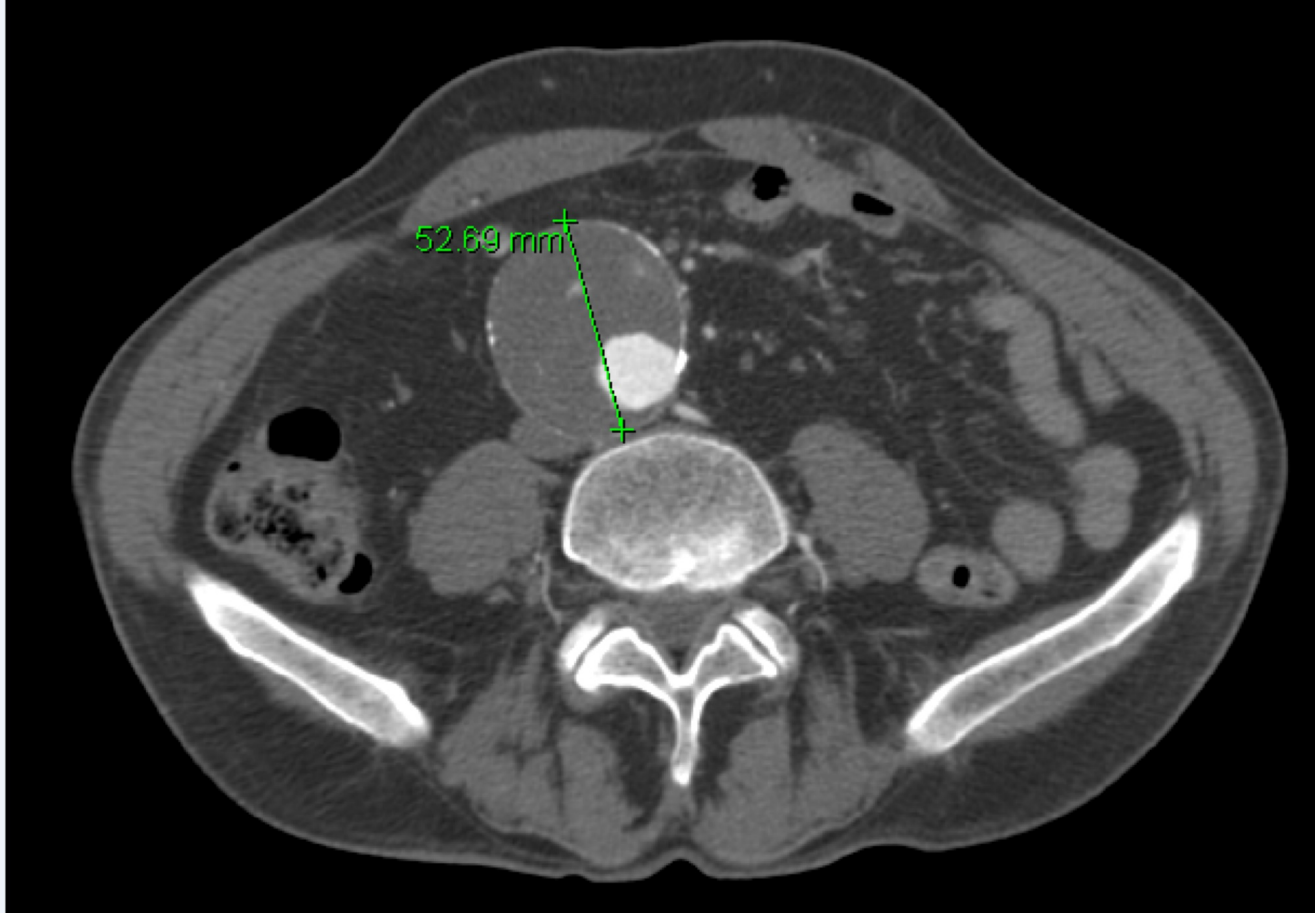
Preoperative abdominal CT angiography demonstrating AAA (green marker) AAA, abdominal aortic aneurysm

**Figure 2:**
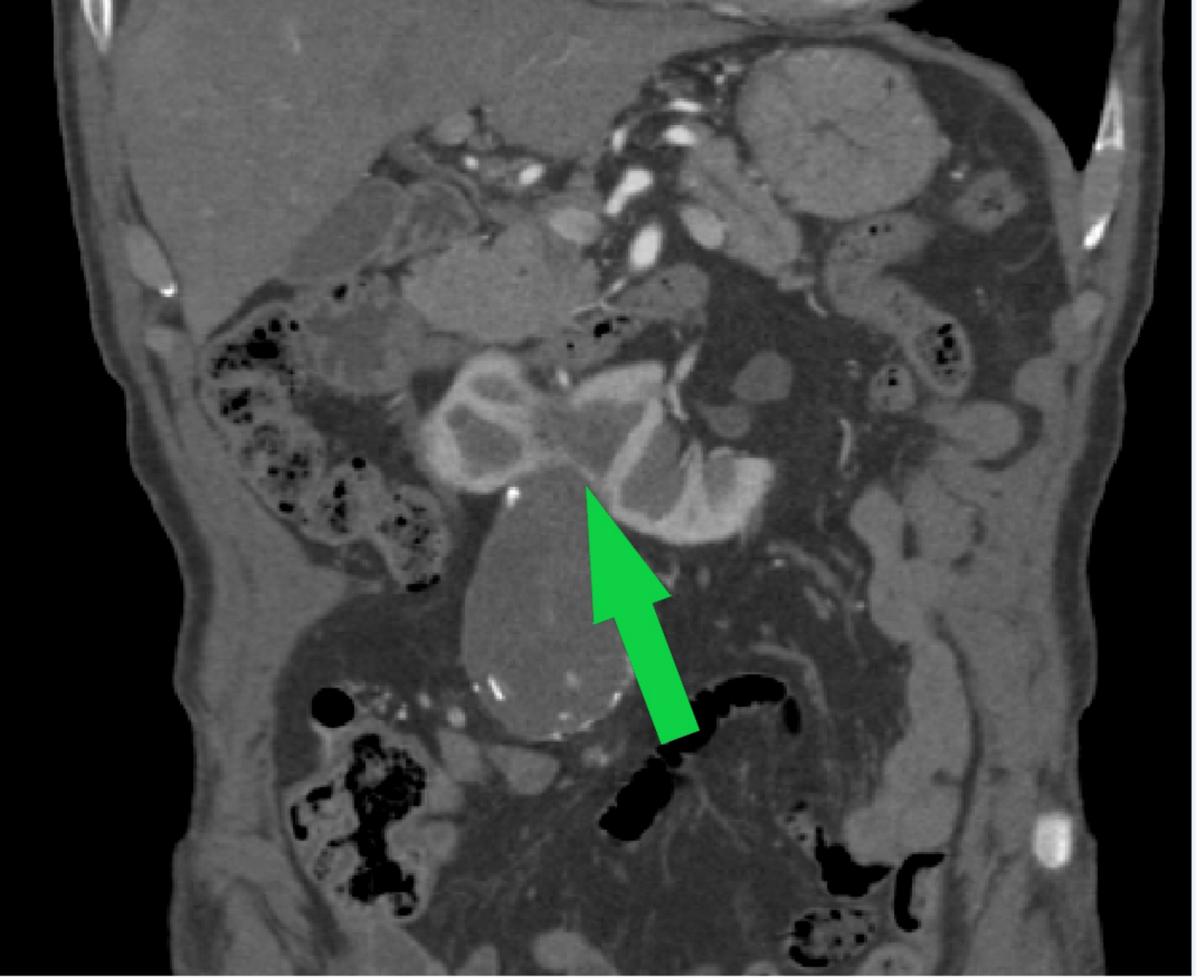
Preoperative abdominal CT angiography, coronal cross-section, demonstrating HSK (green arrow) HSK, horseshoe kidney

An 82-year-old male presented to our vascular surgery clinic as a consultation following a screening abdominal aortic ultrasound that demonstrated an enlarging AAA, 5.6 cm in maximal diameter up from 3.8 cm eight years prior. He was afebrile with a heart rate of 82 beats per minute and a systolic blood pressure of 148 mmHg. On physical examination, he had a body mass index of 21.9 and a palpable abdominal mass. He was in excellent health, endorsing only hypertension for which he took atorvastatin, was a never smoker, and had never had surgery. A decision was made to complete a computed tomography angiography (CTA) scan of his aorta and place him on the Medtronic (Minneapolis, MN) watch list for endovascular consideration. His CTA demonstrated that the AAA was 5.3 in maximal dimension and infrarenal in nature; HSK was also demonstrated (Figures 1, 2).

The patient was deemed an appropriate PEVAR candidate but elected to continue with serial observation at that time. On subsequent follow-up in January 2019, a new abdominal ultrasound demonstrated likely growth of the AAA compared to his previous imaging. Given these findings, he wished to proceed with PEVAR. 

**Figure 3:**
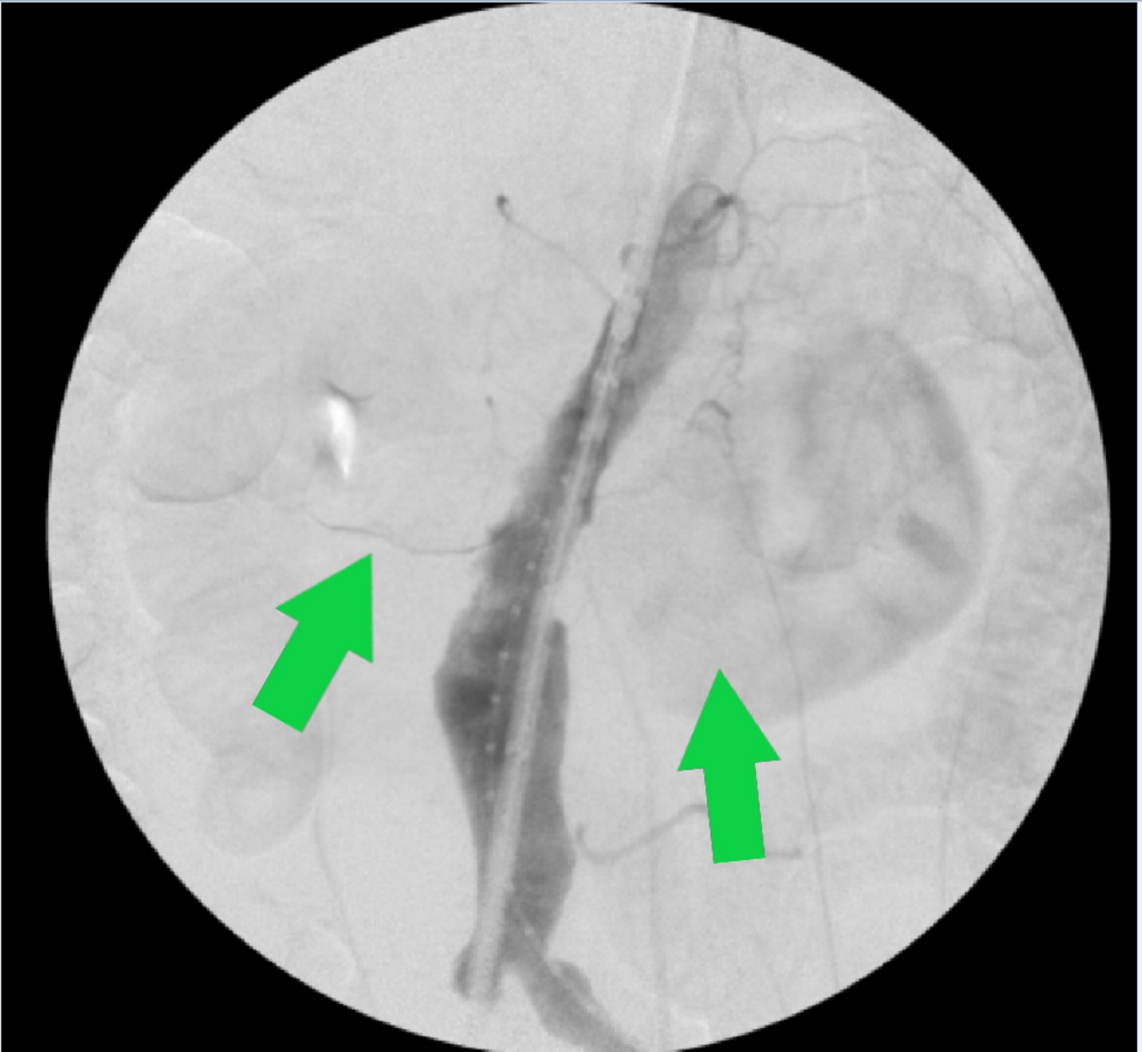
Intraoperative angiogram demonstrating HSK around the AAA (green arrows) AAA, abdominal aortic aneurysm; HSK, horseshoe kidney

He underwent PEVAR with the Medtronic Endurant II stent graft. The patient had easy access into both femoral arteries and preoperative closure devices were placed. A pre-deployment angiogram was performed (Figure 3).

**Figure 4:**
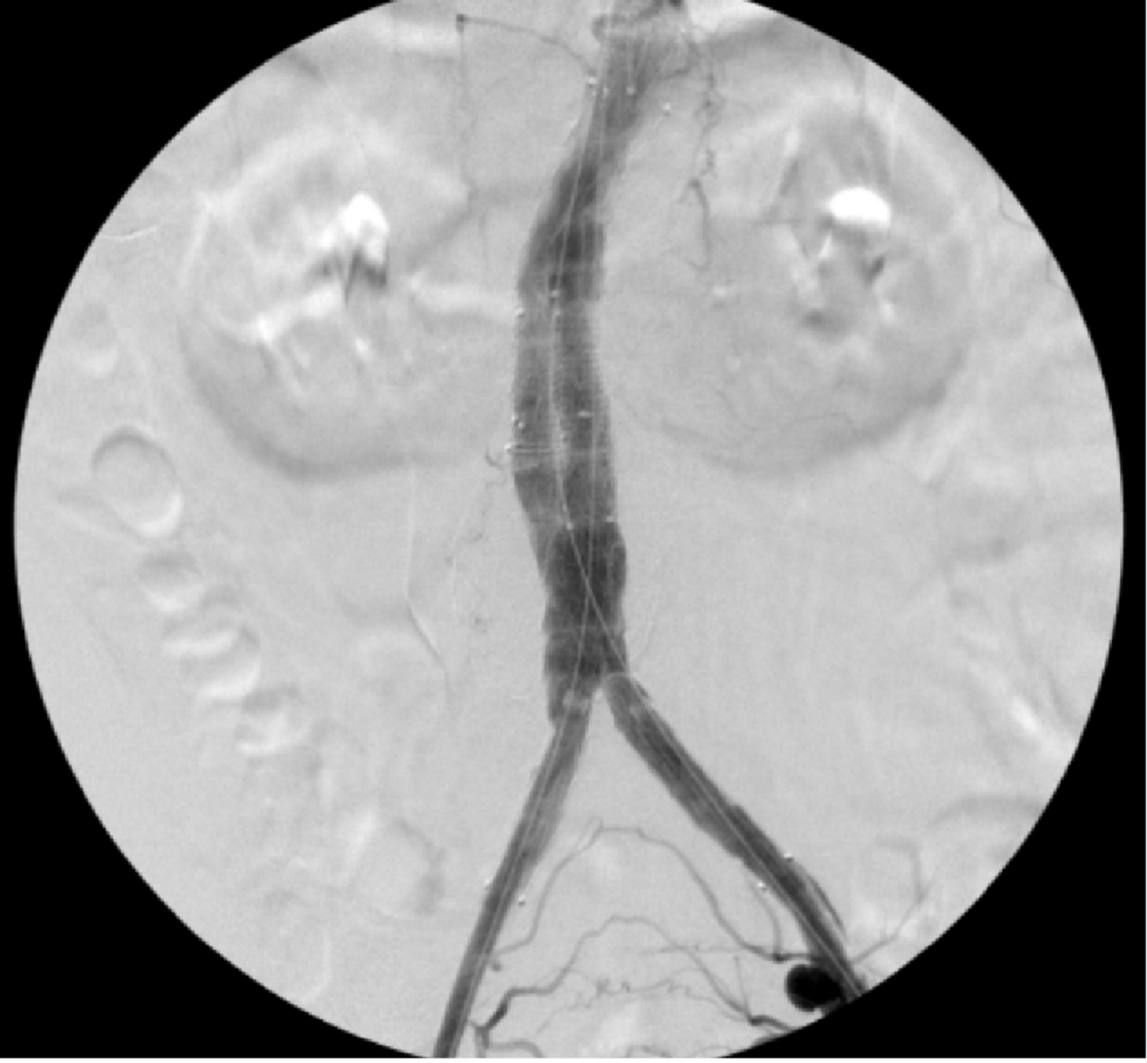
PEVAR completion angiogram showing successful stent placement without contrast extravasation PEVAR, percutaneous endovascular aneurysm repair

The graft was placed below the renal arteries and above the internal iliac arteries. A stenosis in the right iliac artery was noted and multiple balloon angioplasties were done without satisfactory effect, so the decision was made to place kissing stents. During the deployment of these, one of the balloons ruptured while expanding the stent. However, this did not have any effect on the deployment, and the iliac stenosis resolved with good outflow. A completion angiogram did not demonstrate an endoleak (Figure 4).

All wires and sheaths were then removed, and the closure devices utilized to complete the case. He was kept supine for four hours and had an uneventful postanesthesia care unit recovery before being transferred to the ICU for standard postoperative care. His urine output was noted to be bloody, but this cleared over the course of several hours in the setting of appropriate urine output, so his foley catheter was removed.

**Figure 5:**
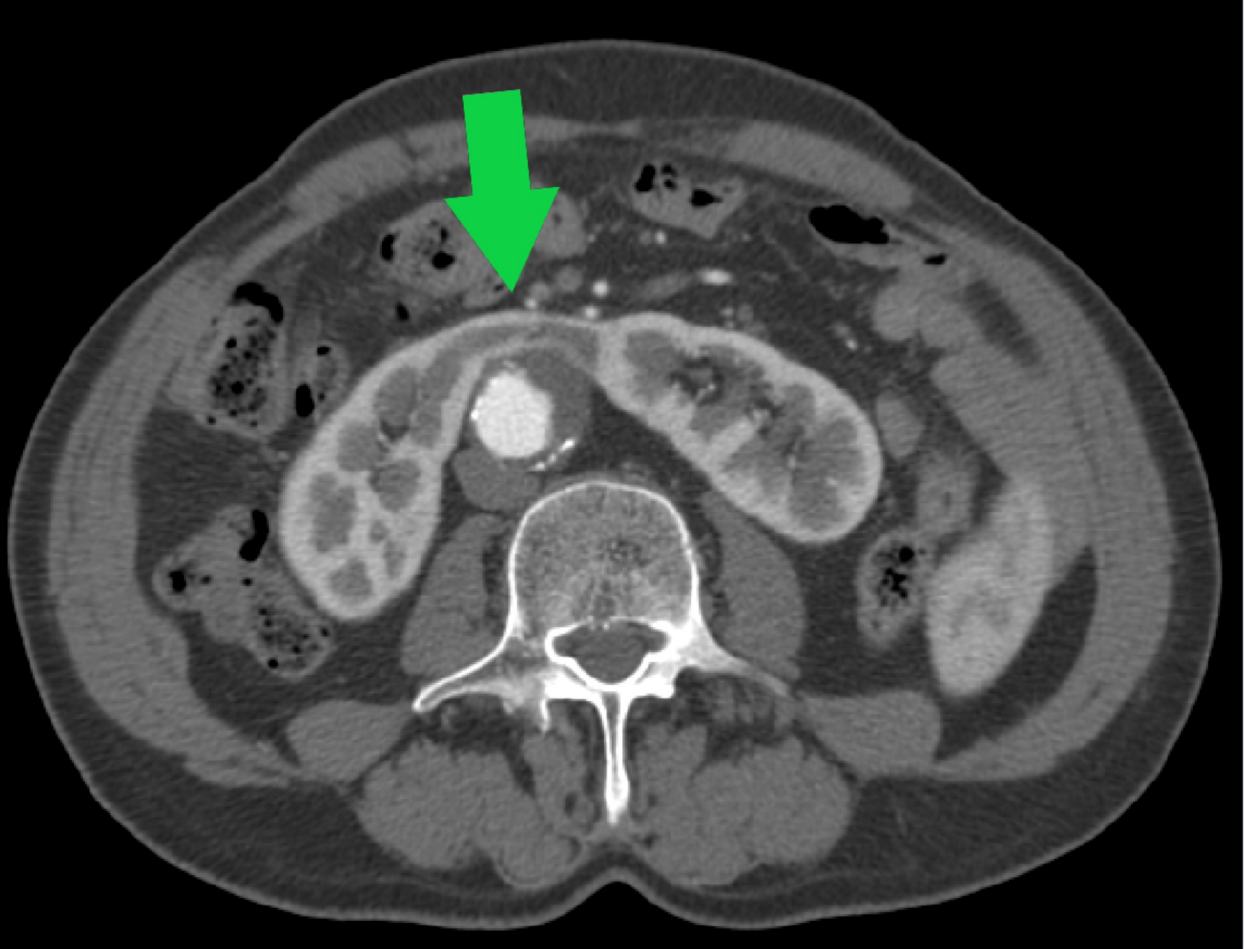
Preoperative abdominal CT angiogram demonstrating appropriate isthmus perfusion (green arrow)

**Figure 6:**
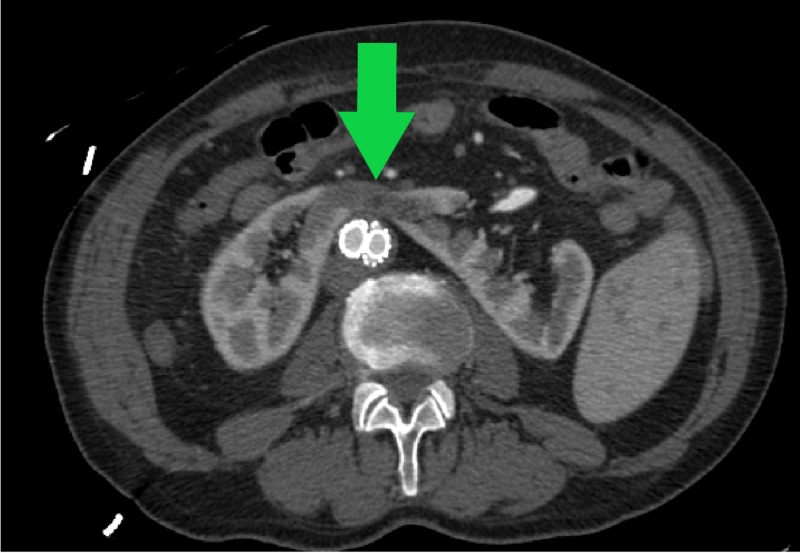
Postoperative abdominal CT angiogram demonstrating hypodense, infarcted HSK isthmus (green arrow) HSK, horseshoe kidney

Later that night he began to experience worsening suprapubic tenderness. A bladder scan demonstrated urinary retention and so a straight catheter was used with good urine output; however, this was without improvement in symptoms, and so his foley was replaced. He was normocardic and normotensive. His groin surgical sites were soft without any hematoma and he had exquisite suprapubic tenderness to palpation. A CTA was obtained and ruled out retroperitoneal hematoma, but did demonstrate infarction of the HSK isthmus, which was noted to be an interval changed compared to the preoperative CTA (Figures 5, 6).

He was maintained on intravenous hydration and given adequate medications for pain control. Nephrology and urology were consulted for further recommendations, and were in agreement that his pain would likely resolve without any further clinical significance to infarction. On postoperative day 2, the patient was transferred out to the stepdown unit, had interval removal of his foley, and was able to be discharged later that day.

**Figure 7:**
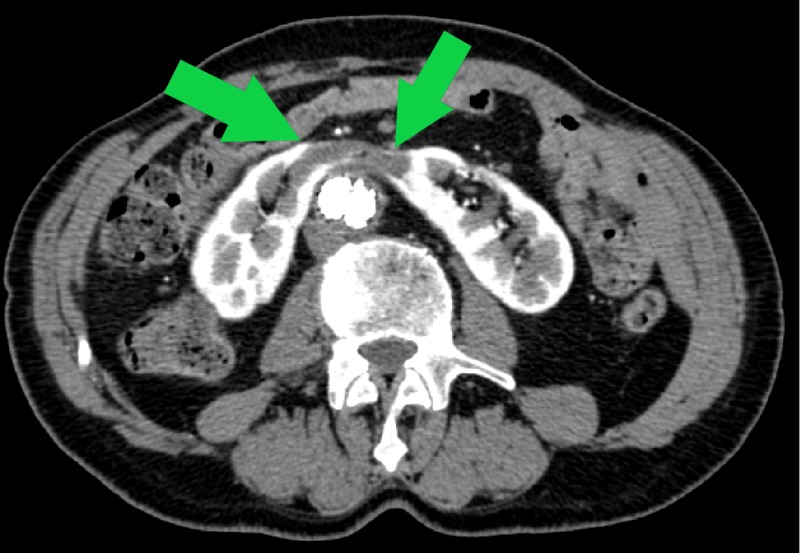
Follow-up imaging demonstrating improved perfusion of the HSK isthmus with complete infarction resolution HSK, horseshoe kidney

He presented to the vascular clinic for routine two-week postoperative follow-up. He complained of bilateral hip pain, suprapubic pressure, and urinary frequency, all of which were slowly improving. He underwent follow-up abdominal CTA which demonstrated stable 5.5 cm AAA status post EVAR with no endoleak, and his HSK as unremarkable, with apparent resolution of the previous isthmus infarction (Figure 7).

He was given reassurance and plan for continued serial postoperative imaging of his repair per local protocol.

## Discussion

HSK is the most common renal fusion defect, estimated to be present in one in 500 individuals [3]. In the majority of these cases, there is an isthmus of parenchymal tissue connecting the two renal pelvises at the inferior poles. This anatomy can lead to some difficulties during open AAA repair, as the presence of the renal isthmus affects both surgical exposure and proximal aortic control. Midline laparotomy provides best exposure to the aneurysm and kidney, but the left retroperitoneal approach avoids interference with the renal isthmus and urinary tracts; however, access to the right iliac artery is limited and difficult. While resection of the isthmus may be necessary to achieve adequate exposure, it may lead to complications such as bleeding and urinary leak. These are complications that can be avoided with EVAR and PEVAR techniques.

The arterial anatomy of HSK can also be quite variable [4]. Graves described a classification system for HSK based on such blood supply, though this may oversimplify the nuances of collateral flow that is often present, even to the contralateral kidney [4,5]. At times the renal arteries supplying the HSK may arise from the aneurysm itself. In open AAA repair, the decision whether or not to reimplant or ligate an accessory renal artery must take into account the patient’s baseline renal function and the associated anatomy. In EVAR, Ruppert et al. have suggested that coverage of accessory renal arteries is safe, especially when their diameter is less than 3 mm and there is no pre-existing renal dysfunction [6].

Workup for AAA in patients with HSK proceeds in the usual fashion with CTA to confirm anatomy and consideration for EVAR/PEVAR based on appropriate anatomy for graft selection. Specifically, imaging needs to assess aortoiliac diameters, lengths, angles, thrombus/occlusive disease, and any aberrant vascular anatomy. Our team was able to identify these factors when planning for the repair of the patient described above, and elected for the safer endovascular approach. As PEVAR has increasingly been associated with significant reductions in operative times, length of stay, surgical site complications, and patient discomfort, our experience is to proceed with PEVAR over EVAR if feasible [7]. Even with a technically successful operation, the HSK anatomy demonstrated to be challenging when the patient developed infarction in the postoperative period. Multidisciplinary discussions between the vascular surgery, urology, and nephrology teams concluded that the most likely etiology was mass effect of the balloon during deployment of the kissing stents. His postoperative course was otherwise uncomplicated, and regular follow-up imaging and examination has not demonstrated any untoward sequelae.

## Conclusions

HSKs present a unique challenge to the vascular surgeon, as their wide-ranging anatomy can complicate open approaches to the aorta for aneurysm repair. Here we report what we believe is the first successfully described PEVAR to treat a patient with HSK. Endovascular options have emerged as the desired approach for AAA treatment when technically feasible, and offer further advantages in the setting of HSK due to the ability to avoid isthmus resection, collecting system damage, or other disturbances to the renal system. Care for these unique patients will continue to improve as surgical technologies and techniques grow over the coming years.
